# A Novel Fluorescent Probe for Hydrogen Peroxide and Its Application in Bio-Imaging

**DOI:** 10.3390/molecules26113352

**Published:** 2021-06-02

**Authors:** Yingying Zuo, Yang Jiao, Chunming Ma, Chunying Duan

**Affiliations:** State Key Laboratory of Fine Chemicals, Dalian University of Technology, Dalian 116024, China; z943877035@mail.dlut.edu.cn (Y.Z.); sxcm98@gmail.com (C.M.); cyduan@dlut.edu.cn (C.D.)

**Keywords:** hydrogen peroxide, reactive oxygen species, fluorescence, imaging

## Abstract

Hydrogen peroxide (H_2_O_2_) plays an important role in the human body and monitoring its level is meaningful due to the relationship between its level and diseases. A fluorescent sensor (**CMB**) based on coumarin was designed and its ability for detecting hydrogen peroxide by fluorescence signals was also studied. The **CMB** showed an approximate 25-fold fluorescence enhancement after adding H_2_O_2_ due to the interaction between the **CMB** and H_2_O_2_ and had the potential for detecting physiological H_2_O_2_. It also showed good biocompatibility and permeability, allowing it to penetrate cell membranes and zebrafish tissues, thus it can perform fluorescence imaging of H_2_O_2_ in living cells and zebrafish. This probe is a promising tool for monitoring the level of H_2_O_2_ in related physiological and pathological research.

## 1. Introduction

Reactive oxygen species (ROS) play important roles in a wide variety of biological functions [1,2], and its abnormal production or accumulation is also closely related to many physiological and pathological processes [3,4], especially, the concentration of ROS in cancer cells is much higher than that in normal cells. Hydrogen peroxide (H_2_O_2_), the least reactive and mildest oxidant among reactive oxygen species [5], can regulate many physiological processes in organisms and even affect the growth and development of cell [6,7,8]. It is produced in low levels during the metabolism of normal living cells and excessive H_2_O_2_ production or accumulation in vivo is also considered as the key contributor to many diseases [9,10,11]. H_2_O_2_ is overproduced when there is an exogenous stimulus or the content of antioxidants is severely reduced, which may be found in numerous diseases, such as cardiovascular disease and cancer [12,13,14]. Thus, it is significant to find a method that can detect and quantify the production of H_2_O_2_ to facilitate the diagnosis and treatment of diseases.

In recent years, various analytical methods that can detect hydrogen peroxide have been reported, such as fluorescence spectroscopy, electroanalysis, chemiluminescence, etc. [15]. The fluorescence detection method is paid much attention among many other analytical methods due to its characteristics of good selectivity, high sensitivity, quick response rate, as well as real-time detection [16,17,18]. It tends to be used for detecting and tracking certain biomolecules in cells or organisms [19] because of its non-invasiveness and ease of operation [20,21,22], and thus has great potential in detecting small molecules’ markers to help in the diagnosis of some diseases. Some fluorescent small-molecule probes for detecting ROS have been reported [23,24,25,26,27]. These studies proved the feasibility of using fluorescent small-molecule probes to detect H_2_O_2_ and can promote the development and progress of H_2_O_2_ probes, which may facilitate the realization of its applications in medicine.

**Scheme 1:**
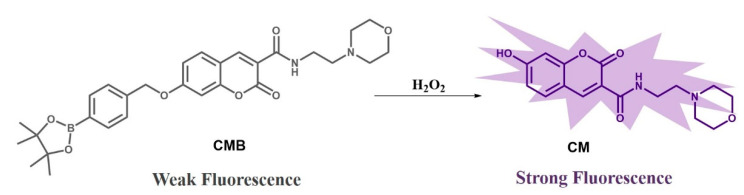
The mechanism of **CMB** to detect H_2_O_2_.

Herein, a smart small-molecule fluorescent probe, **CMB**, was designed and synthesized for H_2_O_2_ imaging in solution, cells, and zebrafish by employing coumarin as fluorophore and the boronate as the recognition group [28,29,30] (Scheme 1). A short alkyl chain is used to connect the morpholine group with coumarin to improve the biological applicability of the probe. The fluorescence signal of **CMB** would be enhanced in the presence of H_2_O_2_ due to the reaction between probe **CMB** and H_2_O_2_, and the good linear relationship between the fluorescence intensity and the concentration of H_2_O_2_ also facilitates its detection. Probe **CMB** can not only shows enhanced fluorescence signal to H_2_O_2_ with good selectivity, but also exhibits desirable imaging effects on H_2_O_2_ in cells and zebrafish, which indicates that **CMB** has the potential to monitor the content and level of hydrogen peroxide in biological systems.

## 2. Results and Discussion

### 2.1. Design Strategy of Probe ***CMB***

**Scheme 2:**
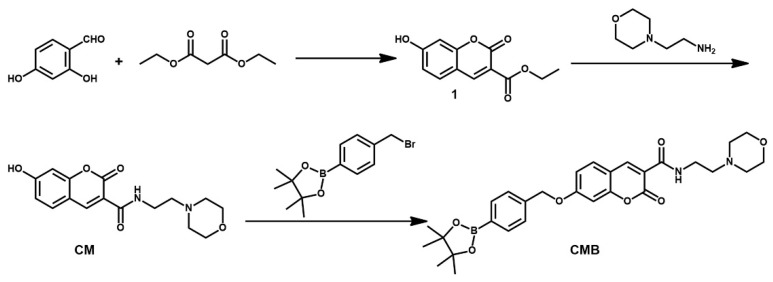
The synthesis route of probe **CMB**.

The probe **CMB** was designed and synthesized in three steps from the coumarin moiety (Scheme 2) that possesses excellent photo-physical properties [31,32]. The morpholine group can increase the solubility of the probe in water, thereby increasing biocompatibility and making it more beneficial for imaging in biological environments. The aryl boronate can be bound to the hydroxyl group of **CM** by the ether linkage strategy to form **CMB** and react with H_2_O_2_ as well as providing the specificity for H_2_O_2_ over other interference species [33,34]. The synthetic route of probe **CMB** was provided and shown in Scheme 2. The structure of **CMB** was accurately validated using the ^1^H NMR and HRMS analyses presented in the Supporting Information (Supplementary Figures S1–S4).

### 2.2. Optical Response of ***CMB*** to H_2_O_2_

**Figure 1:**
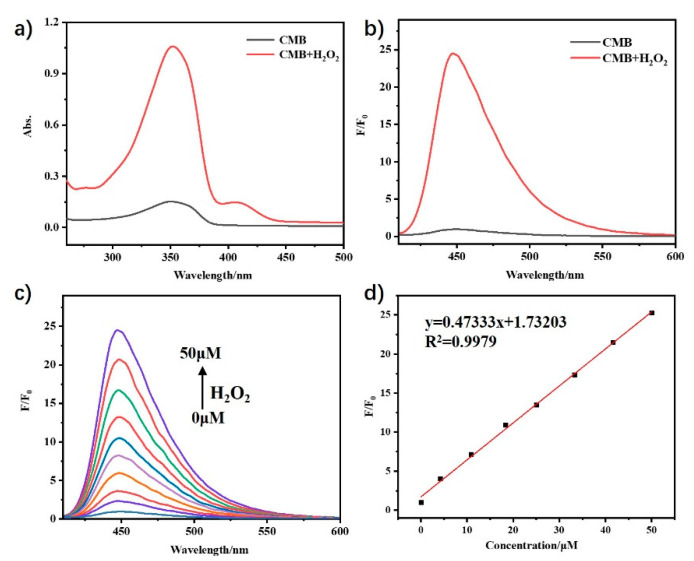
(**a**) UV-vis absorption and (**b**) fluorescence emission spectra of **CMB** before and after addition of H_2_O_2_. (**c**) Fluorescence responses of **CMB** (2 μM) to various concentrations of H_2_O_2_. (**d**) The linear correlation between the fluorescence intensity at 450 nm and H_2_O_2_ concentration.

The UV-vis absorption and fluorescence emission spectra were both studied in acetonitrile/phosphate buffer (1:9 *v*/*v*, 10 mM, pH 7.4) at room temperature to test the optical properties of **CMB** in the presence or absence of H_2_O_2_. The UV-vis spectrum of the probe **CMB** (6 μM) showed a absorption band at 350 nm, then a new band appeared at 405 nm after being treated with H_2_O_2_ (Figure 1a), which may be attributed to the chemical structure change from **CMB** to **CM**. As shown in Figure 1b, **CMB** had only a very weak fluorescence when excited at 400 nm, while a significant increase in fluorescence was observed at 450 nm after adding H_2_O_2_. With the increase of hydrogen peroxide concentration, the fluorescence intensity of **CMB** increased by about 25-fold (Figure 1c). There is a good linear relationship between the fluorescence intensity and the concentration of H_2_O_2_ in the range of 0 to 50 μM with a correlation coefficient of 0.9979 (Figure 1d) and the detection limit was 0.13 μM, thus being able to be used for direct imaging of physiological H_2_O_2_ [23]. This fluorescence change may be attributed to the structural change from **CMB** to **CM** caused by the reaction with H_2_O_2_. The good “off–on” response of **CMB** toward H_2_O_2_ showed its potential for detection of H_2_O_2_ and may realize the detection of H_2_O_2_ level.

**Figure 2:**
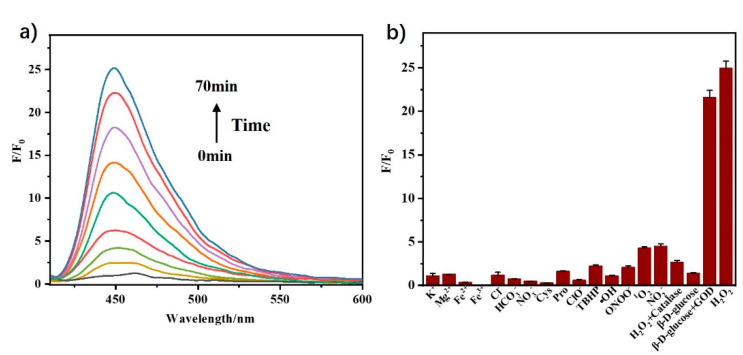
(**a**) The time response of the probe **CMB**. (**b**) Fluorescence response of **CMB** (2 μM) to various competing species (100 μM), including K^+^, Mg^2+^, Fe^2+^, Fe^3+^, Cl^−^, HCO_3_^−^, NO_3_^−^, cysteine (Cys), proline (Pro), hypochlorite (ClO^−^), tert-butyl hydroperoxide (TBHP), hydroxyl radical (•OH), peroxynitrite (ONOO^−^), singlet oxygen (^1^O_2_), nitrite (NO_2_^−^), H_2_O_2_ + catalase, β-D-glucose, β-D-glucose + glucose oxidase (GOD), and H_2_O_2_.

The response time of probe **CMB** toward H_2_O_2_ was also investigated by the time-dependent fluorescence intensity. After adding H_2_O_2_ to the solution of probe **CMB**, the fluorescence intensity at 450 nm gradually increased with time and stabilized finally. The selectivity study of probe CMB was carried out in the presence of various biologically relevant possible competing species, mainly including ions (K^+^, Mg^2+^, Fe^3+^, Cl^−^, etc.), amino acids (Cys, Pro), reactive oxygen species (•OH, TBHP, ClO^−^, etc.) [35,36]. As shown in Figure 2b, ions, amino acids, and most of the reactive oxygen species showed negligible changes in fluorescence and ^1^O_2_ and NO_2_^−^ caused a slight response. The fluorescence enhancement of **CMB** to H_2_O_2_ is more obvious relative to the interferences. In order to further verify the specific reaction between the probe and H_2_O_2_, the catalase (hydrogen peroxide quencher) and glucose oxidase (hydrogen peroxide producer) were used to perform the fluorescence experiment. When H_2_O_2_ is treated with catalase, **CMB** no longer produced a significant increase in fluorescence toward it, but when glucose reacted with glucose oxidase was added, the obvious fluorescence enhancement was observed due to the H_2_O_2_ produced by the reaction, which proved the specificity of **CMB** to H_2_O_2_. The obvious fluorescence response and extended linear range as well as good biocompatibility and permeability make **CMB** an excellent candidate and can serve as the indicators for H_2_O_2_ detection compared with other candidates (Supplementary Table S1). All the results illustrated that **CMB** can react with hydrogen peroxide selectively to achieve fluorescence enhancement and we hope that it is suitable in a bio-environment.

### 2.3. Response Mechanism of ***CMB*** to H_2_O_2_

**Figure 3:**
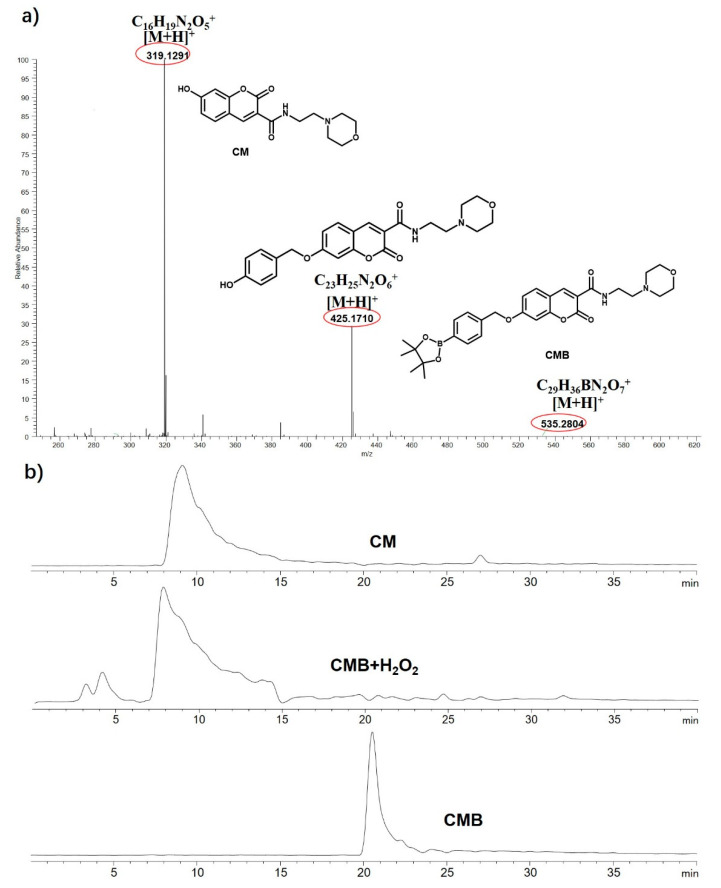
(**a**) HRMS of **CMB** after adding H_2_O_2_. (**b**) Liquid chromatography of **CMB, CMB** treated with H_2_O_2_, and **CM**.

According to the fluorescence response of **CMB** to H_2_O_2_, the possible response mechanism of H_2_O_2_ detection can be preliminarily assumed (Scheme 1). When the fluorophore was connected with the aryl boronate, **CMB** showed almost no fluorescence due to the intramolecular interaction. The added H_2_O_2_ will react with the aryl boronate group, causing the hydroxyl of the fluorophore to be released, thereby revealing a strong fluorescent signal. In order to confirm the mechanism, high-resolution mass spectrometry (HRMS) was performed. **CMB** showed an intense peak at *m*/*z* = 535.2606 (Supplementary Figure S4). After adding H_2_O_2_, the peak of **CMB** had almost disappeared, while a new peak at *m*/*z* = 319.1291 appeared accompanied by the peak of the intermediate product at *m*/*z* = 425.1710, proving the transformation of **CMB** into **CM** by the reaction of aryl boronate group and H_2_O_2_. The liquid chromatography experiments were also done. As shown in Figure 3b, the **CMB** had a signal peak at 20.528 min. After adding H_2_O_2_, a new peak appeared at 9.116 min, which had the same as the retention time of **CM**. Thus, the experiments both supported the fact that the structural transformation caused by the reaction of **CMB** with H_2_O_2_ triggers the enhanced fluorescence signal, proving our inference about the response mechanism of **CMB** to H_2_O_2_ proposed in Scheme 1.

### 2.4. Cytotoxicity and Fluorescence Imaging in Living Cells

**Figure 4:**
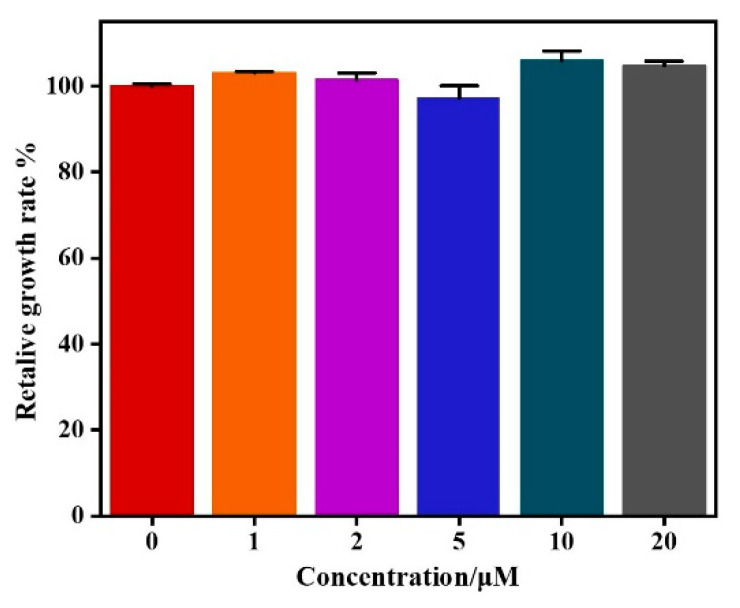
Relative growth rate (%) of cells that were cultured in the presence of a 0 to 20 μM probe **CMB** for 24 h and estimated by the CCK8 assay.

Taking into account the particularity of the biological environment, the viability of cells against the probe was determined using the CCK8 assay technique [37] to evaluate the cytotoxicity of the **CMB**. The relative growth rate of cells was detected upon exposure to **CMB** with concentrations of 0 to 20 μM for 24 h. As is shown in Figure 4, the cell survival rate was greater than 90% at probe concentrations up to 20 μM, demonstrating that **CMB** has an acceptable cytotoxicity to cells within this concentration range and can be used in biological systems. Thus, it can perform the subsequent cell imaging experiment.

**Figure 5:**
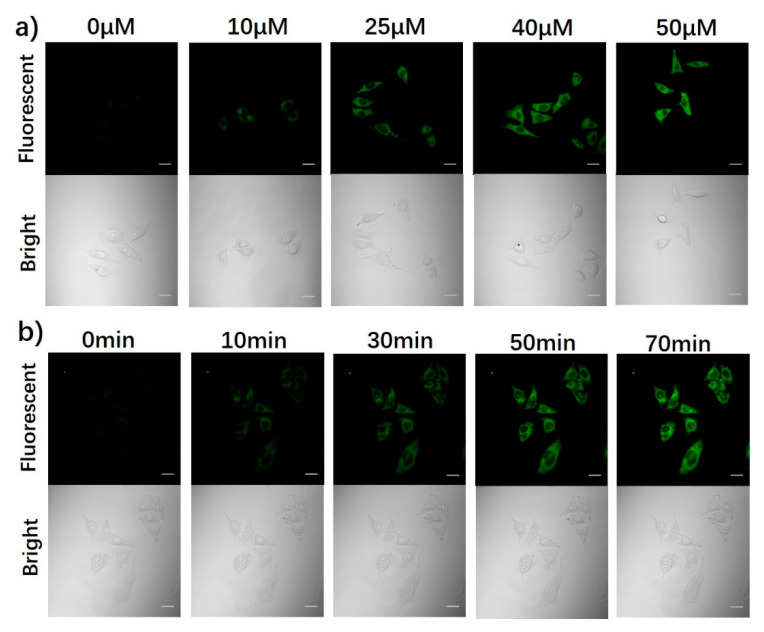
Confocal images after the addition of exogenous H_2_O_2_ in MCF-7 cells. (**a**) Cells were stained with different concentrations of H_2_O_2_ and then incubated with **CMB** (2 μM). (**b**) Cells were pretreated with H_2_O_2_ (50 μM), and then incubated with **CMB** (2 μM) at different time points (0 to 70 min). The excitation wavelength was 405 nm and the emission was collected at 420 to 520 nm. Scale bar = 20 μm.

Then, we carried out the imaging experiment to test the practical feasibility of **CMB** for exogenous and endogenous H_2_O_2_ detection. When MCF-7 cells were stained with **CMB** alone, there was almost no fluorescence, but was observed that the fluorescence signal increased significantly and dose-dependently after treating cells with different concentrations of H_2_O_2_ (0 to 50 μM) (Figure 5a), which also proved that the **CMB** could effectively penetrate the cell membrane and disperse in the cytoplasm. In addition, the cells were incubated with H_2_O_2_ and then treated with **CMB** for imaging at different time points. It was found that the fluorescence intensity gradually increased over time (Figure 5b), which further indicates that **CMB** can be used to detect exogenous H_2_O_2_.

**Figure 6:**
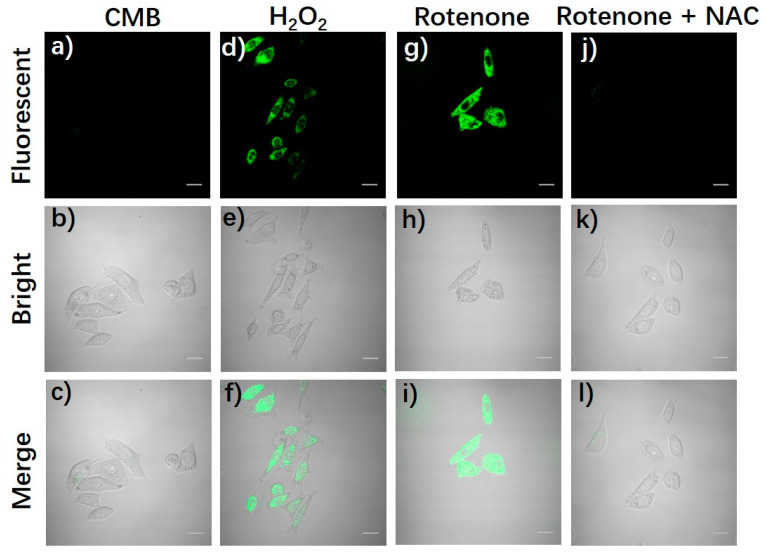
Confocal images for exogenous and endogenous H_2_O_2_ in MCF-7 cells. (**a**–**c**) Cells were stained with **CMB** (2 μM). (**d**–**f**) Cells were incubated with H_2_O_2_ and then stained with **CMB** (2 μM). (**g**–**i**) Cells were incubated with rotenone and then stained with **CMB** (2 μM). (**j**–**l**) Cells were incubated with rotenone, treated with NAC, and then stained with **CMB** (2 μM). The excitation wavelength was 405 nm and the emission was collected at 420 to 520 nm. Scale bar = 20 μm.

In addition, endogenous hydrogen peroxide is generated by the stimulation of rotenone [38,39], so the imaging ability of **CMB** to the endogenous H_2_O_2_ level of MCF-7 cells could be studied by adding rotenone. When cells were treated with rotenone with the addition of **CMB**, an obvious fluorescence could be observed (Figure 6). After incubating the rotenone-pretreated cells with *N*-acetylcysteine (NAC) [40], a common H_2_O_2_ inhibitor, the intracellular fluorescence was basically negligible, indicating the H_2_O_2_ produced by the stimulation of rotenone was eliminated. These results proved the ability of **CMB** to detect endogenous H_2_O_2_. The cell imaging experiment demonstrated that the **CMB** has favorable membrane-permeability, showing its potential for fluorescence imaging of exogenous and endogenous H_2_O_2_ levels in cells.

### 2.5. Zebrafish Imaging

**Figure 7:**
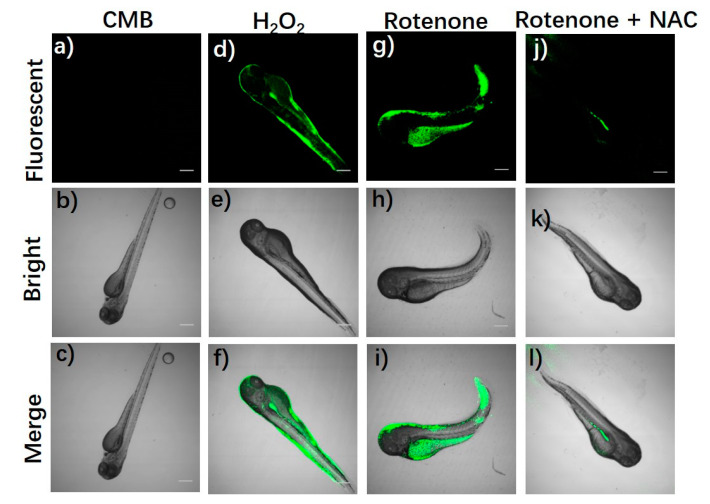
Confocal images for exogenous and endogenous H_2_O_2_ in zebrafish. (**a**–**c**) Zebrafish were stained with **CMB** (2 μM). (**d**–**f**) Zebrafish were incubated with H_2_O_2_ and then stained with **CMB** (2 μM). (**g**–**i**) Zebrafish were incubated with rotenone and then stained with **CMB** (2 μM). (**j**–**l**) Zebrafish were incubated with rotenone, treated with NAC, and then stained with **CMB** (2 μM). The excitation wavelength was 405 nm and the emission was collected at 420 to 520 nm. Scale bar = 300 μm.

To further investigate the availability of the probe **CMB** in biological systems, a zebrafish imaging experiment was carried out to detect its fluorescence imaging performance. As shown in Figure 7, zebrafish incubated with H_2_O_2_ and then treated with **CMB** showed obvious fluorescence, while the control group treated only with **CMB** exhibited negligible fluorescence. Besides, the fluorescence intensity of zebrafish treated with rotenone also increased significantly, and after further treatment with NAC, its weak fluorescence was similar to that of the control group. In addition, the z-scan mode of the confocal microscope was used to record the fluorescence intensity at different depths in the body of the zebrafish to explore the penetration capability of the probe in tissue. As shown in Supplementary Figure S5, bright fluorescence was observed even at a depth of up to 180 μm, showing the probe **CMB** had tissue penetrating and staining capabilities. Thus, **CMB** has excellent penetration and exhibits good imaging feasibility for endogenous and exogenous H_2_O_2_ in zebrafish.

## 3. Conclusions

In summary, a novel fluorescence probe **CMB** based on coumarin was designed and developed successfully, that could interact with H_2_O_2_ and realize its detection by fluorescence. It can react with H_2_O_2_ selectively, thus producing an enhanced fluorescence signal. The probe **CMB** has low toxicity and good biocompatibility, and it can penetrate the cell membranes and zebrafish tissues to image endogenous and exogenous H_2_O_2_. The good penetration and staining ability further prove the feasibility of **CMB** to accurately monitor H_2_O_2_ in biological systems. It is expected that **CMB** will become a promising tool to detect the H_2_O_2_ and exhibits important potential applications in the diagnosis of H_2_O_2_-related diseases.

## 4. Materials and Methods

### 4.1. Materials and Instruments

All chemicals used were of reagent grade or purchased as high-grade commercial products and used without further purification. Solvents used were purified via standard methods. ^1^H NMR spectra were recorded on a Bruker AVANCE III 400 MHz spectrometer. Mass spectrometric data were performed on an LTQ Orbitrap XL spectrometer. The high-performance liquid chromatography (HPLC) experiment was recorded on an Agilent 1260 LC system (USA). Fluorescent spectra were achieved with an F7000 fluorescence spectrophotometer. The UV–vis spectra were recorded on a TU 1900 UV–vis spectrometer. The Dulbecco’s modified Eagle’s medium (DMEM) that was added with 10% fetal bovine serum (FBS), was used to culture all the cell lines. The OD values of the CCK8 assay were measured by BIO-RAD xMark microplate spectrophotometer. Confocal fluorescence imaging was performed on an OLYMPUS FV1000 confocal microscopy.

### 4.2. Synthesis of Compound ***1***

Compound **1** was synthesized according to a previously published article [41]. 2,4-dihydroxybenzaldehyde (1.38 g, 10.0 mmol) and diethyl malonate (1.92 g, 12.0 mmol) were dissolved in anhydrous EtOH, and then an appropriate amount of piperidine was added. The reaction mixture was refluxed for 12 h and then cooled. The precipitate was collected and washed with cold EtOH to obtain the yellow compound **1** (1.87 g) with a yield of 80%. ^1^H NMR (400 MHz, DMSO-*d*_6_) δ 11.07 (s, 1H), 8.65 (s, 1H), 7.73 (d, *J* = 8.6 Hz, 1H), 6.82 (dd, *J* = 8.6, 2.1 Hz, 1H), 6.70 (d, *J* = 1.9 Hz, 1H), 4.26 (q, *J* = 7.1 Hz, 2H), 1.30 (t, *J* = 7.1 Hz, 3H).

### 4.3. Synthesis of Compound ***CM***

Compound **1** (0.41 g, 2.0 mmol) was dissolved in ethanol, then *N*-(2-methylamino)-morpholine (0.31 g, 2.4 mmol) and triethylamine (2.4 mmol) were added. The mixture was heated to reflux for 8 h and cooled after the reaction was complete. Then the precipitate was filtered, and the filtrate was extracted with dichloromethane and water. The organic layers were combined and dried over anhydrous sodium sulfate and column chromatography (CH_2_Cl_2_:CH_3_OH = 100:1) to obtain compound **CM** (0.417 g, yield 65.4%). ^1^H NMR (400 MHz, DMSO-*d*_6_) δ 11.03 (s, 1H), 8.85 (t, *J* = 5.3 Hz, 1H), 8.79 (s, 1H), 7.82 (d, *J* = 8.6 Hz, 1H), 6.88 (dd, *J* = 8.6, 2.2 Hz, 1H), 6.84–6.73 (m, 1H), 3.74–3.54 (m, 4H), 3.43 (q, *J* = 6.2 Hz, 2H), 2.48 (d, *J* = 6.4 Hz, 2H), 2.42 (s, 4H).

### 4.4. Synthesis of Compound ***CMB***

**CM** (0.32 g, 1.0 mmol) and K_2_CO_3_ (0.42 g, 3.0 mmol) were dissolved in 5 mL CH_3_CN and then 2-(4-(bromomethyl)phenyl)-4,4,5,5-tetramethyl-1,3,2-dioxaborolane (0.42 g, 1.4 mmol) was also added to the mixture. The reaction mixture was heated at 80 °C for 24 h and cooled to room temperature. Then, the mixture was diluted with CH_2_Cl_2_ and water and the aqueous layer was extracted with CH_2_Cl_2_. The combined organic phase was washed with water and dried with anhydrous sodium sulfate, then purified by column chromatography (CH_2_Cl_2_:CH_3_OH = 100:1) to obtain a white solid (0.25 g) with the yield of 46.7%. ^1^H NMR (400 MHz, DMSO-*d*_6_) δ 8.85 (d, *J* = 3.8 Hz, 2H), 7.92 (d, *J* = 8.7 Hz, 1H), 7.72 (d, *J* = 7.8 Hz, 2H), 7.49 (d, *J* = 7.8 Hz, 2H), 7.18 (d, *J* = 2.1 Hz, 1H), 7.12 (dd, *J* = 8.7, 2.2 Hz, 1H), 5.32 (s, 2H), 3.59 (t, *J* = 4.6 Hz, 4H), 3.44 (q, *J* = 6.1 Hz, 2H), 2.47 (d, *J* = 6.4 Hz, 2H), 2.42 (s, 4H),, 1.30 (s, 12H).

### 4.5. Preparation of Related Species and Configuration of Solutions

Probe **CMB** was dissolved with DMSO to prepare stock solutions. Adding 4 μL probe stock solution into 2 mL acetonitrile/phosphate buffer (1:9 *v*/*v*, 10 mM, pH 7.4) with the final concentration of probe as 2 μM for fluorescent spectra test. H_2_O_2_, ClO^−^, TBHP, NO_2_^−^, and NO_3_^−^ obtained from commercial sources were diluted or dissolved in water. Other reactive oxygen species are prepared according to the literature [42,43]. ONOO^−^ was prepared by mixing pre-cooled 0.6 M NaNO_2_, 0.6 M HCl, and 0.7 M H_2_O_2_ into 1.5 M NaOH at 0 °C. Manganese dioxide was then added to the solution to eliminate the residual H_2_O_2_ and was removed by filtration. Its concentration was estimated by its extinction coefficient of 1670 M^−1^ cm^−1^ at 302 nm. A singlet oxygen solution was prepared by the reaction of H_2_O_2_ and sodium hypochlorite solution. Hydroxyl radical was generated by the Fenton reaction and can be acquired by adding Fe^2+^ to H_2_O_2_.

### 4.6. Measurement of Detection Limit

The detection limit was obtained from the fluorescence titration curve and was calculated according to the following equations [44]:

Detection limit = 3σ/k

Here σ represents the deviation of blank and k is the slope of the linear regression equation.

### 4.7. Cell Culture and Cytotoxicity Assay

The Michigan Cancer Foundation-7 (MCF-7) cells were allowed to culture for 24 h at 37 °C with medium supplemented with 10% fetal bovine serum (FBS) in a 5% CO_2_ humidified incubator. Cells were seeded in 96-well microplates for 24 h and then cultured in medium with 0, 1, 2, 5, 10, and 20 μM of probe **CMB** for 24 h. Cells in culture medium without probe were used as the control. The solution of CCK-8 reagent was added to each well and the plates were incubated for another 1 h. The medium was then removed carefully, and the absorbance of solutions was determined on a microplate reader at 450 nm.

### 4.8. Cell Imaging

The MCF-7 cells were treated with different concentrations of H_2_O_2_ (0 μM, 10 μM, 20 μM, 30 μM, 40 μM, 50 μM) and then incubated with **CMB** (2 μM) at 37 °C to detect exogenous H_2_O_2_. In addition, MCF-7 cells were stimulated with rotenone (2 µM) at 37 °C to produce endogenous H_2_O_2_, and then treated with **CMB** (2 µM). Then, another group of MCF-7 cells was first stimulated with rotenone (2 µM), incubated with NAC (1 mM) to eliminate the endogenous H_2_O_2_, and treated with **CMB** (2 µM) at 37 °C. The cells were washed three times with PBS after incubation, and then imaged on a confocal laser microscope with a 60× objective lens.

### 4.9. Zebrafish Imaging

Zebrafish embryos were maintained at 28.5 °C. First, the zebrafish were only incubated with **CMB** (2 μM) as the control group. Another group of zebrafish was stimulated with rotenone (2 µM), and then treated with **CMB** (2 µM). Then the last group of zebrafish was stimulated with rotenone (2 µM), incubated with NAC (1 mM) to eliminate the endogenous H_2_O_2_, and treated with **CMB** (2 µM). The zebrafish were washed three times with PBS after incubation and imaged on a confocal laser microscope with a 4× objective lens.

## Supplementary Materials

The following are available online, Figure S1: The 1H NMR of compound **1** in DMSO-d6 solution, Figure S2: The 1H NMR of compound **CM** in DMSO-d6 solution, Figure S3: The 1H NMR of compound **CMB** in DMSO-d6 solution, Figure S4: The HRMS of compound **CMB**, Table S1: Properties of the previously developed fluorescent H2O2 probes and the probe CMB, Figure S5: The confocal z-scan images of zebrafish treated with probe and H2O2. Zebrafish were incubated with H2O2 and then stained with **CMB** (2 μM). The excitation wavelength was 405 nm and the emission was collected at 420 to 520 nm.

## Sample Availability

Samples of the compounds are not available from the authors.
