# Supplementary material for: A Novel Fluorescent Probe for Hydrogen Peroxide and Its Application in Bio-Imaging
Source: Molecules. 2021 Jun 2;26(11):3352. doi: 10.3390/molecules26113352 (PMC8199646; doi:10.3390/molecules26113352)
Supplement: Supplementary file 1 [file molecules-26-03352-s001.zip › molecules-1226285-supplementary.pdf]

Supplementary Information

A Novel Fluorescent Probe for Hydrogen Peroxide and its Application in Bio-imaging

Yingying Zuo [1], Yang Jiao [1,*], Chunming Ma [1] and Chunying Duan [1]

1. State Key Laboratory of Fine Chemicals, Dalian University of Technology, Dalian 116024, China;

E-mail: jiaoyang@dlut.edu.cn

Content

Figure S1. ^1H NMR of compound 1

Figure S2. ^1H NMR of compound CM

Figure S3. ^1H NMR of compound CMB

Figure S4. ESI-MS spectrum of CMB

Table S1. Properties of the previous developed fluorescent H_2O_2 probes and the probe CMB.

Figure S5. The confocal z-scan images of zebrafish

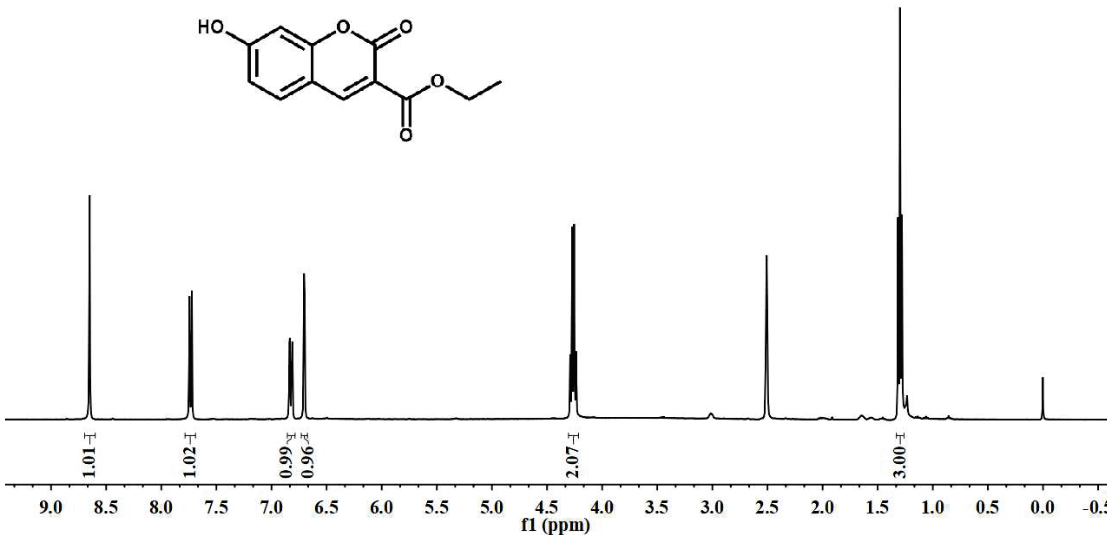

Figure S1. The ^{1}H NMR of compound 1 in DMSO-d_6 solution.

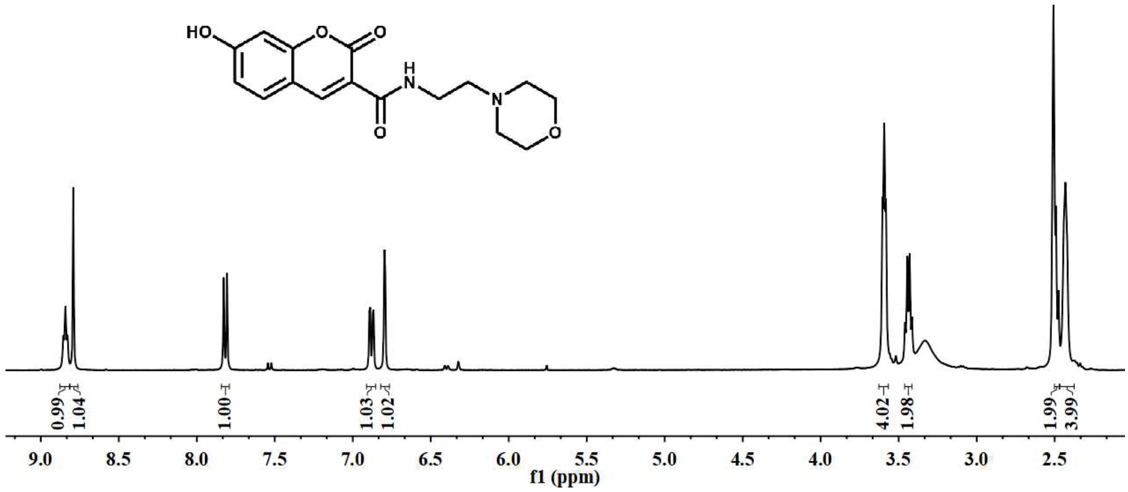

Figure S2. The ^{1}H NMR of compound **CM** in DMSO-d_6 solution.

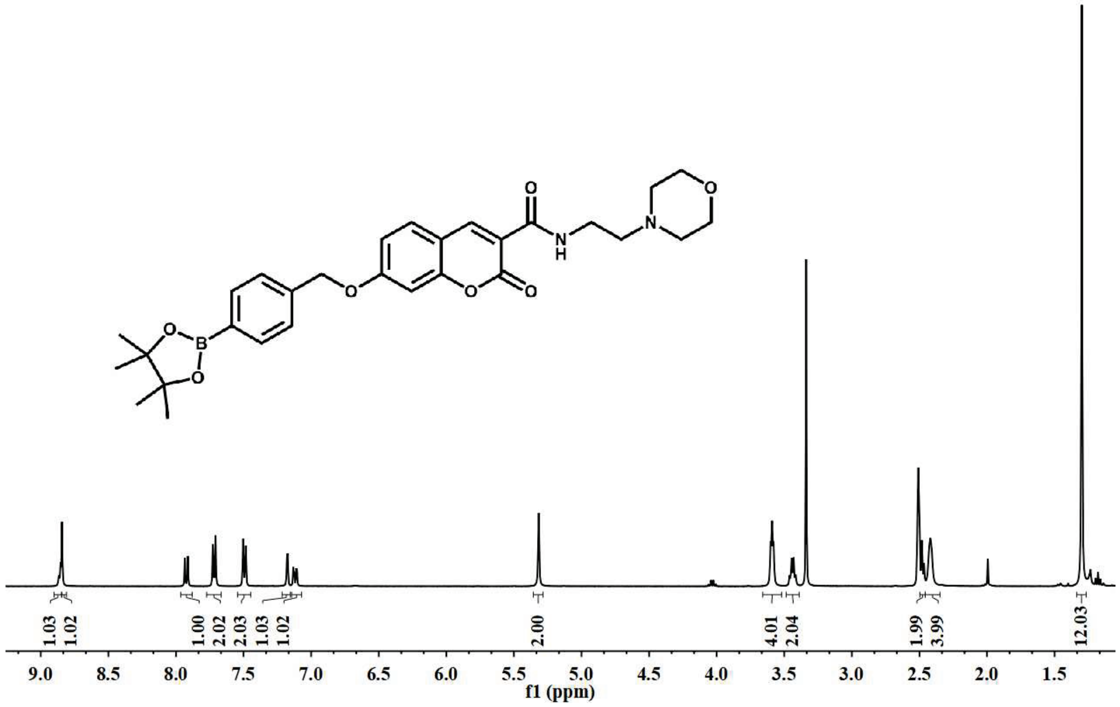

Figure S3. The ^{1}H NMR of compound **CMB** in DMSO-d_6 solution.

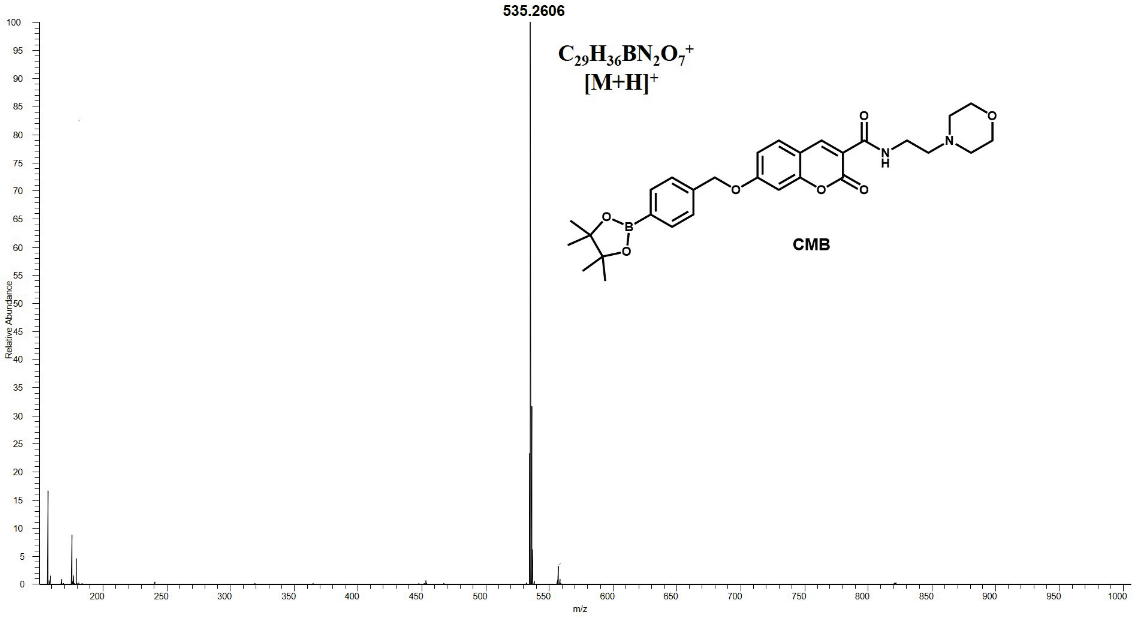

Figure S4. The HRMS of compound **CMB**.

Table S1. Properties of the previously developed fluorescent H_2O_2 probes and the probe **CMB**.

Probe	Synthesis step	Response time/min	Enhance-ment	Linear range/μM	Detection limit	Imaging application	Ref.
LHX-B-CPT	6	80	10	0-140	0.026 μM	Hela cells	[1]
Pyp-B	2	4	8	0-40	0.021 μM	A549 cells and zebrafishes	[2]
BODIPY-Se	3	5	3	0-80	0.13μM	MCF-7, HepG2 cells and zebrafishes	[3]
DCX-B	8	No	13	0.1-100	0.033μM	HepG2, HCT11 cells and mouse	[4]
BTFMB	3	45	20	0-20	109 nM	HepG2 cells	[5]
PB1	3	200	4	0–100	1.38 μM	HepG2 cells and drosophila	[6]
CMB	3	70	25	0-50	0.13 μM	MCF-7 cells and zebrafishes	This work

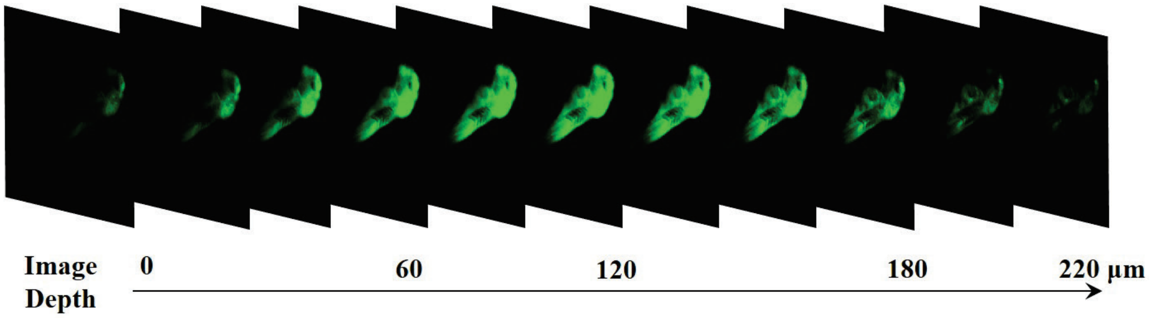

Figure S5. The confocal z-scan images of zebrafish treated with probe and H_2O_2. Zebrafish were incubated with H_2O_2 and then stained with **CMB** (2 μM). The excitation wavelength was 405 nm and the emission was collected at 420–520 nm.

References

1. Liu, J.; Cao, C., A Simple and Effective "Elimination" Approach for Selective Cancer Therapy to Reveal the Role of H_2O_2. *ACS Omega* **2020,** *5*, 22157-22162. https://doi.org/10.1021/acsomega.0c02240.
2. Wang, K.; Ma, W.; Xu, Y.; Liu, X.; Chen, G.; Yu, M.; Pan, Q.; Huang, C.; Li, X.; Mu, Q.; Sun, Y.; Yu, Z., Design of a novel mitochondria targetable turn-on fluorescence probe for hydrogen peroxide and its two-photon bioimaging applications. *Chinese Chemical Letters* **2020,** *31*, 3149-3152. https://doi.org/10.1016/j.cclet.2020.08.039.
3. Xu, C.; Qian, Y., A selenamorpholine-based redox-responsive fluorescent probe for targeting lysosome and visualizing exogenous/endogenous hydrogen peroxide in living cells and zebrafish. *J Mater Chem B* **2019,** *7*, 2714-2721. https://doi.org/10.1039/c8tb03010c.
4. Wang, W.-X.; Jiang, W.-L.; Liu, Y.; Li, Y.; Zhang, J.; Li, C.-Y., Near-infrared fluorescence probe with a large stokes shift for visualizing hydrogen peroxide in ulcerative colitis mice. *Sensors and Actuators B: Chemical* **2020,** *320*, 128296. https://doi.org/10.1016/j.snb.2020.128296.
5. Zhang, H.; Tian, D.; Zheng, Y.; Dai, F.; Zhou, B., Designing an ESIPT-based fluorescent probe for

imaging of hydrogen peroxide during the ferroptosis process. *Spectrochim Acta A Mol Biomol Spectrosc* **2021,** *248*, 119264. https://doi.org/10.1016/j.saa.2020.119264.

6. Qiu, X.; Xin, C.; Qin, W.; Li, Z.; Zhang, D.; Zhang, G.; Peng, B.; Han, X.; Yu, C.; Li, L.; Huang, W., A novel pyrimidine based deep-red fluorogenic probe for detecting hydrogen peroxide in Parkinson's disease models. *Talanta* **2019,** *199*, 628-633. https://doi.org/10.1016/j.talanta.2019.03.017.
